# Comparison and optimization of nanoscale extracellular vesicle imaging by scanning electron microscopy for accurate size-based profiling and morphological analysis†

**DOI:** 10.1039/d0na00948b

**Published:** 2021-03-22

**Authors:** Sara Cavallaro, Petra Hååg, Kristina Viktorsson, Anatol Krozer, Kristina Fogel, Rolf Lewensohn, Jan Linnros, Apurba Dev

**Affiliations:** Department of Applied Physics, School of Engineering Sciences, KTH Royal Institute of Technology 10691 Stockholm Sweden saracav@kth.se; Department of Oncology-Pathology, Karolinska Institutet 17164 Solna Sweden; Department of Smart Hardware, Division of Digital Systems, Research Institutes of Sweden AB 40014 Gothenburg Sweden; Theme Cancer, Medical Unit Head and Neck, Lung, and Skin Tumors, Thoracic Oncology Center, Karolinska University Hospital 17164 Solna Sweden; Department of Electrical Engineering, The Ångström Laboratory, Uppsala University 75121 Uppsala Sweden apurba.dev@angstrom.uu.se apurbad@kth.se

## Abstract

Nanosized extracellular vesicles (EVs) have been found to play a key role in intercellular communication, offering opportunities for both disease diagnostics and therapeutics. However, lying below the diffraction limit and also being highly heterogeneous in their size, morphology and abundance, these vesicles pose significant challenges for physical characterization. Here, we present a direct visual approach for their accurate morphological and size-based profiling by using scanning electron microscopy (SEM). To achieve that, we methodically examined various process steps and developed a protocol to improve the throughput, conformity and image quality while preserving the shape of EVs. The study was performed with small EVs (sEVs) isolated from a non-small-cell lung cancer (NSCLC) cell line as well as from human serum, and the results were compared with those obtained from nanoparticle tracking analysis (NTA). While the comparison of the sEV size distributions showed good agreement between the two methods for large sEVs (diameter > 70 nm), the microscopy based approach showed a better capacity for analyses of smaller vesicles, with higher sEV counts compared to NTA. In addition, we demonstrated the possibility of identifying non-EV particles based on size and morphological features. The study also showed process steps that can generate artifacts bearing resemblance with sEVs. The results therefore present a simple way to use a widely available microscopy tool for accurate and high throughput physical characterization of EVs.

## Abstract 2

Nanoscale extracellular vesicles (EVs) have emerged as a valuable source of disease biomarkers. Here, we present a direct visual approach for their accurate morphological and size-based profiling by using scanning electron microscopy.

## Graphical abstract

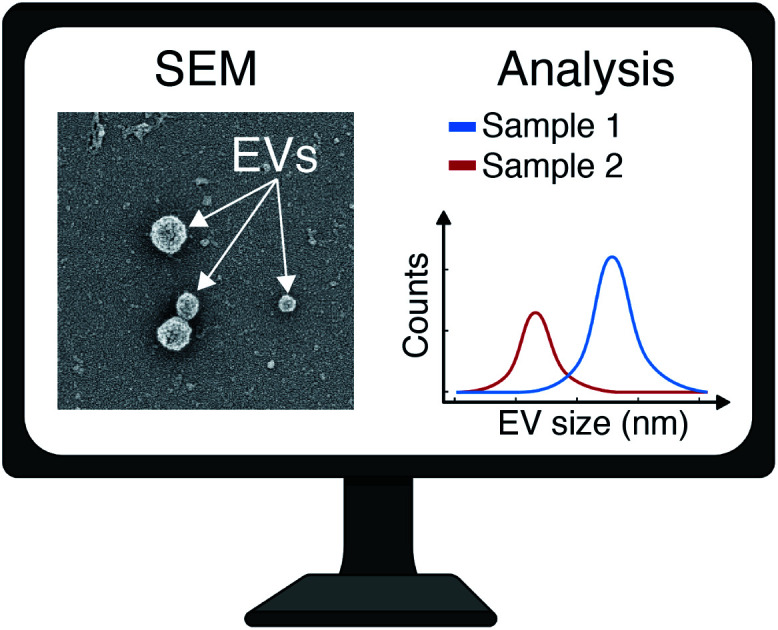

## Introduction

During the past decade, research efforts have significantly enhanced our understanding of extracellular vesicles (EVs), their biological relevance and pathological significance, particularly in cancer.^1–5^ To support this rapidly expanding field, a number of analytical tools such as nanoparticle tracking analysis (NTA), flow cytometry (FC), tunable resistive pulse sensing (TRPS), and dynamic light scattering (DLS) as well as various microscopy methods including electron microscopy (EM, *e.g.* transmission EM, scanning EM, cryo-EM) or atomic force microscopy (AFM) have been proposed for the physical characterization of EVs.^6–8^ These methods have played a major role in the overall development of the field, as physical characterization is essential to optimize various EV isolation and enrichment protocols and benchmark them in terms of purity, concentration and size distribution of the isolated EVs. These characterization methods, however, have very large differences in their accuracy, measurable EV size range and concentration, throughput, analytical time and cost.^9^ A comparison of these features for some of the commonly used EV characterization techniques is presented in Table 1. While NTA and FC, widely used tools in EV characterization, offer the benefit of high throughput and operational simplicity, the methods suffer from a lower resolution and have less capacity to interrogate EVs of smaller size, *i.e.* below 60–70 nm.^6,9,10^ Besides, NTA provides hydrodynamic radii, thus it may overestimate the particle size, and cannot distinguish between a spherical object and a particle of random shape.^9,11,12^ Similar limitations in the detection of small particles and their morphologies also apply to other techniques, *e.g.* tunable resistive pulse sensing (TRPS) and interferometric reflectance imaging sensing (IRIS).^6,10^ Moreover, the recent understanding concerning the heterogeneity in EV populations has introduced a new analytical challenge as well as a renewed interest in high-resolution physical characterization of EVs.^13,14^ Various recent reports not only suggest that populations of EVs exist in the size range of ∼50–60 nm or below,^13,15–18^ but also that EVs are very heterogeneous in their size and shape even when following the same route of biogenesis.^19^ In addition, recent investigations also indicate that the stiffness of EVs is influenced during certain physio-pathological conditions.^20^ This suggests that the shape, deformation and morphology of EVs are likely to become important for further understanding of their biophysical properties and functional heterogeneity. Given the inherent limitations of analysis by scattering-based approaches,^6,9^ these conditions are not likely to be fully met by available technologies such as NTA. This necessitates the development of new methodologies, which are also widely available and inexpensive. Microscopy-based approaches such as transmission EM (TEM), scanning EM (SEM) and AFM offer very high resolution and sensitivity, in addition to providing the possibility to analyze particle morphology. However, these technologies lack standardization and throughput, suffering from low or operator dependent quality and reproducibility.^9,21^ These drawbacks have made many of the images obtained by these techniques very heterogeneous^22–25^ or insufficient for EV size profiling.^26^ Therefore, to be used for reliable and reproducible EV characterization, microscopy-based methods need further improvement and development. Cryo-EM, in this respect, certainly offers a major advantage, but the technique is neither widely available nor cost effective for frequent sample analysis.

**Table 1:** Comparison of widely used EV physical characterization methods. For high accuracy, concentration measurement, morphological information and reproducibility, green ticks indicate that the tool is suitable or has been demonstrated to prove the different monitoring capacities.^10^ Red crosses are used to indicate that the tool is unsuitable or that it has not been demonstrated to provide the element in any publication, to the best of our knowledge, at the time of writing this article. High accuracy refers to the precision of the method in determining the exact size of the particle,^9–11^ and concentration measurement refers to the capability of the method to estimate the EV concentration (in particles per mL) in the analyzed sample.^6,10,27^ Morphological information refers to the capability of the method to discriminate the shape of the measured particle, while reproducibility refers to its capability to show similar results across different studies.^10^ For the entire EV population detection column, ticks depict that the technique is able to detect sizes of 20–1000 nm, while crosses indicate that only a subrange of sizes can be monitored.^9^ For high-throughput, ticks indicate the capability of the method to analyze >5000 particles per sample in ∼10 min, while crosses indicate a lower count rate.^9^ We stated the reproducibility of the IRIS technique as “not available, (NA)” given the novelty of the method and the absence of a significant amount of scientific reports on this technique

Technique	High accuracy	Concentration measurement	Entire EV population detection	Morphological analysis	High throughput	Reproducibility
NTA	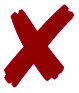	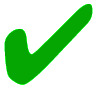	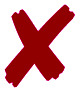	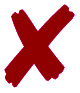	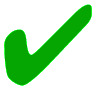	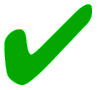
FC	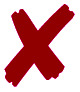	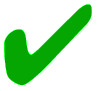	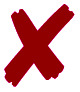	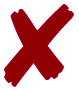	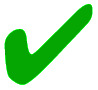	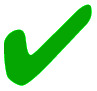
TRPS	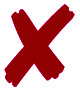	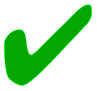	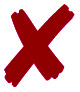	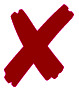	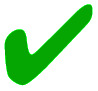	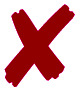
IRIS	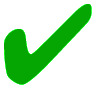	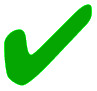	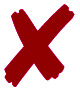	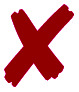	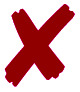	NA
SEM	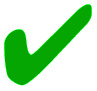	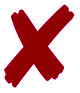	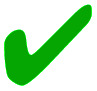	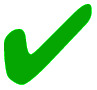	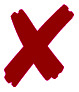	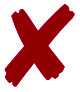
TEM/Cryo-TEM	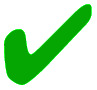	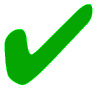	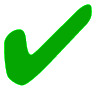	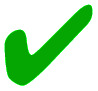	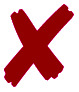	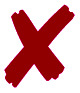
AFM	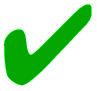	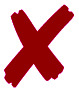	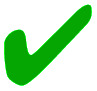	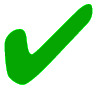	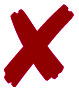	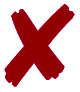

In this report, we demonstrate the prospect of using scanning electron microscopy for high resolution size-based profiling of EVs with improved throughput. Our focus on SEM was based on the fact that it provides morphological information about particles and represents a lower proportion of EM studies on EVs as compared to TEM. Moreover, the SEM technique has a higher throughput than AFM. In particular, here we present a comparison of different pre-imaging steps and substrate functionalization protocols for improvement of throughput and image quality. The study was performed on both cell-line derived sEVs, isolated from a non-small-cell lung cancer (NSCLC) cell line, and sEVs isolated from human serum. We tested different preparation protocols for SEM with NSCLC cell line derived sEVs and analyzed their influence on image quality and throughput. We then applied the optimal parameters to profile serum sEVs isolated by two different methods, namely size exclusion chromatography (SEC) and tangential flow filtration (TFF). The comparison of sEV size distributions obtained by SEM and NTA revealed that while the distribution of larger sEVs (diameter > 70 nm) closely followed each other, these two methods significantly deviated for smaller sized sEVs. The SEM based approach clearly identified a higher proportion of EV like particles in a smaller size range (<70 nm). In addition, we also identified the presence of particles of random shapes within the reported sEV size range from the analyzed samples, indicating the importance of morphological analysis for accurate EV identification and size profiling.

## Materials and methods

### Reagents

High purity deionized water (DIW) with a resistivity of 18 MΩ cm was used throughout all the experiments. Phosphate-buffered saline (PBS, P4417) in tablets and a Grade I glutaraldehyde solution (G5882) specifically purified for EM were purchased from Sigma-Aldrich. If not stated otherwise, all the other chemicals were purchased from Sigma-Aldrich and filtered using a 0.45 μm filter before use.

### EV isolation

The EVs investigated in this study were collected from two different sources and isolated in order to obtain small EVs (sEVs, 30–300 nm). The sEVs used to develop the SEM protocol were isolated from the conditioned cell culture medium of the NSCLC cell line H1975 (ATCC, LGC Standards, Wesel, Germany) and isolated using qEVoriginal size exclusion chromatography columns (SEC, IZON, Oxford, UK). For sEV isolation, the H1975 cells were grown in RPMI-1640 medium supplemented with exosome-depleted FBS (#Gibco™ A2720801, Thermo Fisher Scientific, Stockholm, Sweden). The medium, which was collected after 48 h of cell culture, was centrifuged at 200 g for 5 min followed by 2000 g for 10 min. Around 50 mL medium was concentrated to about 500 μL using Amicon® Ultra-15 Centrifugal Filter concentrators with a 3k cutoff (Merck Chemicals and Life Science AB, Solna, Sweden). The qEVoriginal columns were rinsed with 15 mL PBS (0.22 μm filtered) and the samples were added followed by gradual addition of PBS. Fractions of 500 μL were collected and the main sEV containing fractions without protein contamination (fractions 6–10 according to the company) were pooled and concentrated using Amicon® Ultra-4 Centrifugal Filter concentrators (Merck Chemicals and Life Science AB).

sEVs used to validate the platform were collected from commercial human serum from healthy individuals, which was purchased from Merck Millipore (#S1-100 mL, lot 3163484, the same lot in all preparations) and isolated using two different methods, namely SEC on qEVoriginal columns and tangential flow filtration (TFF), respectively. According to the manufacturer, the serum sample was filtered through a conventional 220 nm filter prior to freezing and subsequent shipment. The frozen serum sample was thawed and used within 24 hours. For sEVs isolated *via* SEC, the same protocol as for NSCLC cell culture medium isolated sEVs presented above was followed. However, as a first step, the serum sample was concentrated from 4 mL to around 700 μL using Amicon® Ultra-4 Centrifugal Filter concentrators with a 3k cutoff. For sEVs isolated *via* TFF, MicroKros filters from Spectrum Labs (now Replingen) were used in pore sizes 200 nm and ∼20 nm (500 kD). Prior to use, all filters were rinsed with MilliQ water to remove glycerol, which is used by the manufacturer to preserve pore sizes (according to manufacturer description). Thereafter, filters and all tubing were blocked by a 10 mg mL^−1^ solution of bovine albumin in PBS, which was allowed to permeate through the pores for at least 3 hours, and then flushed with PBS. A serum sample of 20 mL was circulated through the MicroKros filter system for 4 h and thereafter a retentate of 4 mL was obtained, containing particles larger than about 20–30 nm (500 kD) and smaller than 200 nm.

### EV characterization

sEVs were characterized by NTA for concentration and size estimation and by western blot (WB) for their expression of the tetraspanin proteins CD9 and CD63. NTA measurements were performed on a NS300 instrument (NanoSight, Malvern Panalytical, Malvern, UK), using a 488 nm laser. Serum sEVs were diluted 1 : 200 in PBS and analyzed with the following settings: syringe pump speed 100, detection threshold 8, camera level 13 and analysis time 5 × 60 s. Cell line sEVs were diluted 1 : 100 in PBS and analyzed as follows: syringe pump speed 100, detection threshold 4, camera level 14 and analysis time 3 × 60 s. sEVs isolated from the cell culture media of NSCLC cells as well as from human serum by SEC or TFF were analyzed for CD9 and CD63 expressions using WB. EVs were lysed in 5× RIPA buffer to a final volume of 1× RIPA, sample buffer was added, and lysates ran on a Bis Tris gel, 4–12% with MES buffer (Fisher Scientific). Proteins were transferred to nitrocellulose membranes, blocked with 1 : 1 Odyssey blocking buffer (LI-COR GmbH, Bad Homburg, Germany): TBST, and incubated with respective primary antibodies: anti-CD9 (#13403) and anti-calnexin (#2433) (both from Cell Signaling Technology, BioNordica AB, Stockholm, Sweden) and anti-CD63 (MAB5048) from R&D Systems (Abingdon, UK). Signals from the secondary antibody goat anti-rabbit IRDye® 800CW (LI-COR) were visualized using the Odyssey® Sa Infrared Imaging System (LI-COR).

### Functionalization protocols

For all the protocols, Si substrates (1 cm × 1 cm) with a thermally grown SiO_2_ layer were used. The substrates were cleaned with acetone, isopropanol and DIW in sequence. Three different functionalization protocols, as described in the following section, were investigated.

#### I. Covalent protocol

SiO_2_ substrates were functionalized using our previously reported functionalization protocol up to the glutaraldehyde (GA) step.^28^ Briefly, SiO_2_ wafers were first cleaned in a 5 : 1 : 1 solution of DIW, H_2_O_2_ and NH_4_OH (88 °C, 10 min) and activated with (3-aminopropyl)triethoxysilane (APTES, 5% v/v in 95% ethanol, 10 min) and GA (1% v/v in 1× PBS, 1 h). Thereafter, sEVs were covalently immobilized on top of GA for 1 h, using the GA-amine interaction. Following sEV capture, the remaining GA active sites were deactivated with Tris–ethanolamine (Tris–ETHA, 0.1 M Tris buffer and 50 mM ethanolamine, pH 9.0, 30 min) and casein (0.05% w/v in 1× PBS, 1 h). Finally, the functionalized substrates were washed with 1× PBS prior to the subsequent pre-imaging steps.

#### II. Non-covalent protocol

SiO_2_ substrates were prepared following our previously reported functionalization protocol.^28^ Briefly, after the GA step, the CD9 antibody (50 μg mL^−1^ solution in 1× PBS; ab195422 from Abcam) was immobilized on top of the substrate for 2 h. The antibody targeted the extracellular part of the CD9 transmembrane protein which is known to be expressed in analyzed NSCLC cell sEVs.^28,29^ Thereafter, the remaining GA active sites were deactivated using Tris–ETHA and casein. After 1× PBS washing, sEVs were incubated on top of the antibody-coated substrate for 1 h. Following sEV binding, the substrates were washed with 1× PBS to remove unbound vesicles.

#### III. Control protocol

SiO_2_ substrates were prepared following all the steps of the covalent protocol except for sEV incubation. Briefly, following the GA step, Tris–ETHA and casein were used to deactivate the substrate. Finally, the functionalized substrates were washed with 1× PBS.

For the characterization of cell-line derived sEVs, vesicles were immobilized/incubated at a concentration of 3 × 10^9^ particles per mL. For the characterization of serum sEVs, vesicles were immobilized at a concentration of 1 × 10^10^ particles per mL. Unless stated otherwise, all sEVs were fixed in a solution of GA/paraformaldehyde (PFA) (0.1% GA and 2% PFA in 1× PBS) for 30 min.

### Pre-imaging steps

The pre-imaging steps consisted of sample drying, either in air or by critical point drying (CPD), and sputtering. The influence of these pre-imaging steps was investigated in different combinations. In the case of air drying, substrates with immobilized sEVs were thoroughly washed with water after washing with PBS, in order to minimize crystal formation, and left to dry in air in a fume hood. In the case of CPD, substrates were first dehydrated with a series of ethanol washing cycles (20%, 40%, 60%, 80%, 100%, 10 min each) and then inserted into the CPD for liquid exchange (CO_2_) and drying. Depending on the availability, an E3100 CPD system from Quorum Technologies or an EM CPD300 CPD system from Leica was used, keeping the drying parameters constant.

For the substrates that underwent sputtering prior to SEM imaging, the process was performed in a Polaron SC7640 Au/Pd sputter (Uppsala University). This metal layer was sputtered on top of the samples to increase the lateral conductivity for the SEM electron beam. The sputtering parameters were set in order to deposit an ∼10 nm layer of Au/Pd on top of the substrates.

### SEM imaging and analysis

SEM imaging was performed on an Ultra 55 SEM microscope from Zeiss, using an Inlens detector, with a working distance between 2 mm and 3 mm and a 20 μm aperture. Pixel averaging was set as a noise reduction method. Moreover, the corners of the substrates were connected to the metallic sample holder using a piece of conductive Cu tape, in order to reduce charging effects and improve the image resolution. SEM images were acquired at random locations on the substrates and the collected scans were examined by visual inspection for morphological analysis. For size distribution analysis, the Fiji software was used. In particular, after calibration of the image scale, EV-like particles were separated from the background based on contrast, through application of a threshold value. Thereafter, selected particles were filtered based on size and shape, using the following settings: size 200-infinity (nm^2^) and circularity 0.5–1. The diameters of the vesicles included in the set size and circularity ranges were then calculated and extracted using the “Analyze particles” function in Fiji.

## Results

For the evaluation of various preparation, functionalization and isolation protocols, we acquired SEM images by scanning different substrate areas and by analyzing the number, distribution and morphology of the immobilized sEVs. As presented below, we divided the analyzed parameters into two different groups, namely functionalization-related and pre-imaging parameters. The functionalization-related group includes all the steps necessary for the conjugation of sEVs isolated from NSCLC cell culture medium to the substrates, while the pre-imaging group describes the steps performed after substrate functionalization and prior to SEM imaging. The characterization results of NSCLC cell line derived sEVs isolated by SEC and serum sEVs isolated by SEC or TFF are presented in the ESI (Fig. S1†).

### Functionalization-related parameters

**Fig. 1:**
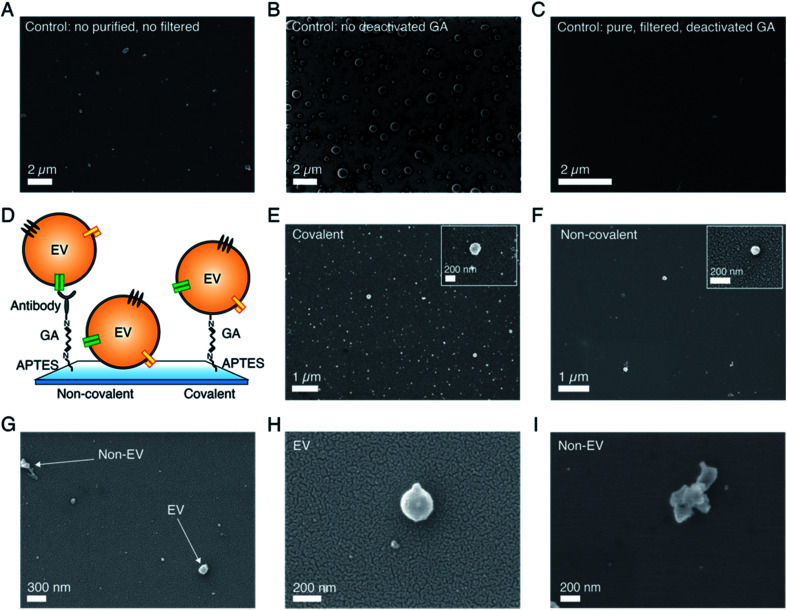
Effects of the functionalization-related parameters on the SEM imaging results for sEVs isolated from the conditioned cell culture medium of NSCLC cells. (A) Control substrate prepared following the control protocol with as-purchased, non filtered functionalization chemicals and a standard GA solution. Accelerating voltage (AV) = 2 kV. (B) Control substrate prepared following the control protocol up to the GA step. No filtered chemicals deposited, and standard GA not deactivated by Tris–ETHA and casein used. AV = 2 kV. (C) Control substrate prepared following the control protocol using filtered chemicals and Grade I and deactivated GA solution. AV = 2 kV. No sputtering used for all control substrates in (A)–(C). (D) Schematic of sEV coupling to a SiO_2_ wafer using non-covalent and covalent captures. Non-covalent coupling occurred *via* antibody capture (non-covalent protocol) or vesicle adsorption, while covalent capture occurred *via* GA–amine interaction (covalent protocol). (E) Representative image of a substrate with sEVs (from the cell culture medium of NSCLC H1975 cells) functionalized following the protocol for covalent capture. Zoomed in image of a few vesicles detected by this strategy. AV = 3 kV. (F) Representative image of a substrate with sEVs (the same source as in (E)) functionalized following the protocol for non-covalent capture, using anti-CD9 antibody. Zoomed in image of a vesicle detected by this strategy. AV = 3 kV. (G) Representative images showing the difference between particles of spherical shape like EVs and particles of random shapes. AV = 3 kV. (H) Zoomed in image of a vesicle-like particle. AV = 3 kV. (I) Zoomed in image of a particle of random shape. AV = 3 kV. An Au/Pd layer (∼10 nm thickness) was sputtered on top of the substrates in (E)–(H). All the samples in this figure were dried using CPD.

To demonstrate the possibility of using SEM for size-based EV profiling and morphological analysis, it is important to identify and reduce artifacts that might come from non-EV particles, surface textures, *etc.*, ultimately resulting in erroneous measurements. Therefore, as a first step, we examined the effects of different functionalization parameters on control substrates, which were prepared by following the control protocol as indicated above. Fig. 1A shows a representative image of a control substrate functionalized by using as-purchased chemicals without any filtration and a standard GA solution. As visible, the substrates showed many particles in the sEV size range (50–200 nm) whose number significantly increased after GA coating (data not shown). Fig. 1B shows instead a representative image of a control substrate prepared using the control protocol up to the GA (standard solution) step (no Tris–ETHA, no casein). As presented, in both cases the controls showed particles in the EV size range that were clearly not vesicles. Such artifacts would also appear in substrates with sEVs, therefore leading to erroneous counts and size distribution measurements. The effect was more pronounced for control substrates where GA was not deactivated by Tris–ETHA and casein (Fig. 1B). In this case, SEM detected many circular particles that could be mistaken as vesicles but were instead created by the GA reaction with some of the chemical compounds used (Fig. 1B). On the other hand, Fig. 1C shows a representative control substrate prepared following the optimized control protocol. In this case, we used a Grade I GA solution specifically purified for EM studies. Furthermore, we filtered all the chemicals prior to deposition, and we deactivated the GA active sites with Tris–ETHA and casein.

As presented in Fig. 1C, the substrates showed the expected results, appearing very clean and with very few particles/objects on top and in the sEV size range. Fig. S2† shows additional representative images of the different control substrates investigated. We emphasize that all the figures presented here depict the general features of the substrates and were verified by randomly analyzing different substrate locations (>10 spots per substrate) and also with multiple substrates prepared by following identical protocols. After having identified and optimized the parameters of the functionalization chemicals that lead to reliable controls, we compared the effects of covalent and non-covalent EV capture. Fig. 1D shows the schematic of these two strategies. In the case of non-covalent capture, we separately conjugated the vesicles to the substrates by antibody coupling and adsorption (Fig. 1D, left and center), whereas for covalent capture, the interaction between GA and amines was used (Fig. 1D, right). As presented in Fig. 1E, F and S3,† the SEM results revealed that the covalent EV strategy retained a larger number of vesicles on the substrates (∼8 particles per μm^2^) as compared to the antibody-based non-covalent one (<1 particle per μm^2^). In this latter case, most of the vesicles were lost during the steps following functionalization due to the low capture strength, resulting in sparsely populated substrates in the SEM images (Fig. 1F). Substrates with vesicles conjugated *via* adsorption showed even lower throughput and thus were not considered in the analysis. Overall, the images showed reasonably good resolution and quality, suggesting that the analyzed sEVs had rather spherical shapes. Moreover, they confirmed the capacity of SEM to distinguish between spherical/oval objects such as vesicles and other particles of random shapes (Fig. 1G–I). The latter could be excluded during image analysis.

### Pre-imaging parameters

**Fig. 2:**
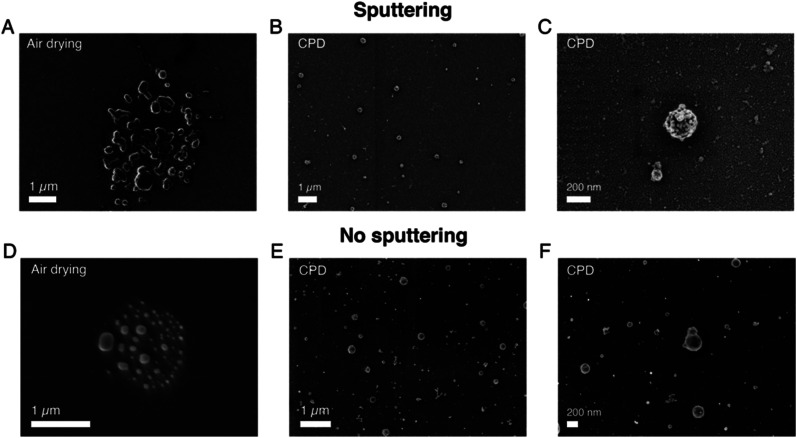
Effects of the pre-imaging parameters on the SEM imaging results of sEVs isolated from the conditioned cell culture medium of NSCLC cells. (A) Representative SEM image of a substrate where sEVs were fixed in a solution of GA/PFA and then dried in air. (B) Representative SEM image of a substrate where sEVs were fixed in a solution of GA/PFA and then dried using CPD. (C) Zoomed in image of a few vesicles dried using CPD. An Au/Pd layer (∼10 nm thickness) was sputtered on top of the substrates in (A)–(C). (D) Representative SEM images of a substrate where sEVs were fixed in a solution of GA/PFA, were dried in air but were imaged without the Au/Pd sputtered layer. (E) Representative SEM images of a substrate where sEVs were dried using CPD but were imaged without the Au/Pd sputtered layer. (F) Zoomed in image of a few vesicles dried using CPD but imaged without the Au/Pd sputtered layer. AV = 1 kV for all images for better resolution of the vesicle surfaces.

As we know, SEM operation requires the application of high vacuum conditions in the microscope chamber and therefore EVs need to be appropriately dehydrated prior to imaging, without affecting their morphology. This is a critical step for biological species, as they contain and/or are surrounded by liquid in their physiological environment. In addition, fixation might also be needed in order to preserve their shapes.^30^ Moreover, the non-conductive nature of biological samples requires the use of a conductive layer on top of them in order to reduce charging effects arising during the electron beam–sample interaction.^31^ Therefore, following functionalization optimization, we analyzed the effects of these different pre-imaging procedures on the final SEM images. Fig. 2 and S4† present the results of the analysis. In particular, first, we analyzed and compared air drying (AD) with critical point drying (CPD), as these are the two commonly used and reported techniques in the EV field.^22–25^

Fig. 2A shows a representative SEM image of sEVs that were dried in air, while Fig. 2B and C show representative SEM images of sEVs that were dehydrated using CPD. Additional images comparing the two drying techniques are presented in Fig. S4.† As is shown, in the case of CPD, vesicles appeared to be uniformly distributed on the substrate and retained their spherical shape (Fig. 2B and C). On the contrary, the vesicles that were dried in air created a “coffee stain effect”,^32^ with distinct islands of clustered particles (Fig. 2A and S4†). Moreover, the images also suggested the presence of substrate areas (Fig. S4†) containing many particles with random sizes and shapes and that the morphology of these vesicles was altered by the drying process. Unlike CPD handled substrates, the vesicles that were dried in air appeared to be more elongated (Fig. 2A and S4†). Although advantageous, CPD suffered from low reproducibility and in some cases did not show the expected results, damaging the substrates. This was possibly due to flaws during the liquid exchange and/or drying process itself. In order to reduce image blurring caused by the charging effect, we acquired the SEM images presented in Fig. 2A–C with a thin (∼10 nm) sputtered conductive layer of Au/Pd. However, this step may induce errors in size estimation, particularly for smaller vesicles. This problem can be avoided by the use of a more conductive substrate. Fig. 2C shows representative SEM images obtained from sEVs that were covalently coupled to a heavily doped Si substrate. As presented, by using conductive substrates, we could still acquire good images of the vesicles, however, the resolution was slightly lower (Fig. 2F) as compared to cases where a thin metal layer was used (Fig. 2C). Furthermore, while the sEVs shown in Fig. 2B and C were fixed in a solution of GA/PFA, the sEVs shown in Fig. 2E and F were not fixed prior to CPD. Similarly, the sEVs shown in Fig. 2A and D were fixed prior to air drying, while the sEVs shown in Fig. S5† were not. Nevertheless, in all the cases the vesicles without fixation showed similar shapes to fixed sEVs treated with the corresponding protocols (CPD or air drying, respectively).

### Application to sEVs isolated from human serum

**Fig. 3:**
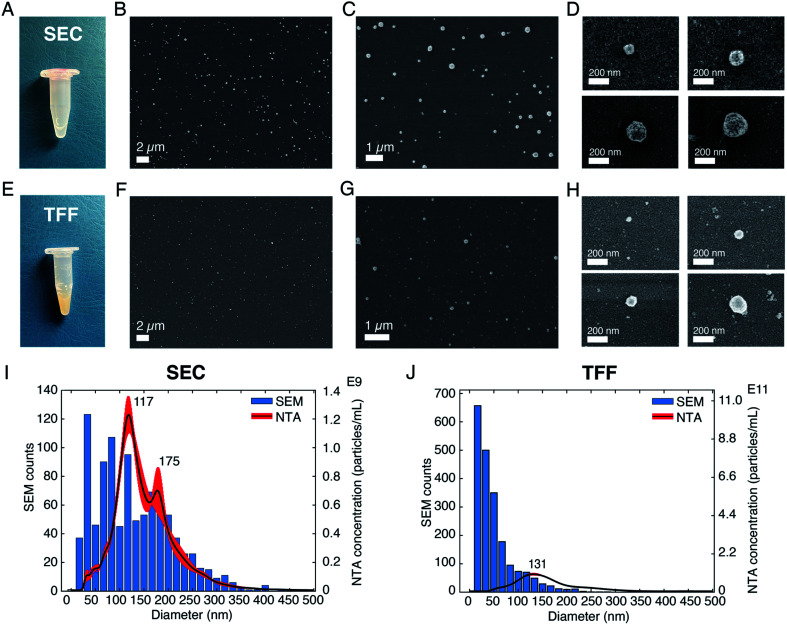
Validation of the SEM protocol on sEVs isolated from human serum. (A) Image of the sEV sample isolated by using a SEC qEVoriginal column. (B)–(D) Representative SEM images of sEVs isolated by SEC, showing vesicles of different diameters. AV = 3 kV for (B) and (C) and AV = 1 kV for (D), for better vesicle surface resolution. (E) Image of the sEV sample isolated by using the TFF technique. (F)–(H) Representative SEM images of sEVs isolated by using TFF. AV = 3 kV for (F) and (G) and AV = 1 kV for (H), for better vesicle surface resolution. (I) Comparison between diameter distributions of the sEVs analyzed by SEM and those analyzed by NTA for the SEC method. (J) Comparison between the diameter distributions of the vesicles analyzed by SEM and those analyzed by NTA for the TFF method.

After having investigated the effects of different parameters on SEM imaging of sEVs isolated from the cell culture medium of NSCLC cells, we applied our optimized protocol to analyze and profile sEVs isolated from healthy human serum. The SEM protocol consisted of (i) the use of pure, filtered and non-reactive chemicals, (ii) covalent sEV capture on the substrate, (iii) fixation and CPD, and (iv) Au/Pd sputtering. Furthermore, to demonstrate the prospect of using SEM for EV size profiling as well as to check the purity of EV isolation techniques, we applied our protocol on sEVs obtained by two different isolation methods, namely SEC and TFF. Fig. 3A–D and E–H show the pictures of the isolated sEV samples and SEM images of sEVs obtained from SEC and TFF based isolation methods, respectively. It has previously been reported that SEC allows for isolation of sEVs with minor contamination of proteins/small particles as compared to TFF.^33^ The latter cannot totally exclude small particles, despite being able to enrich samples within a tailored size window (in our case particles in the size range 30–200 nm). This can also be observed in the images presented in Fig. 3, S6 and S7.† As shown by the pictures of the collected samples (Fig. 3A and E), the sEVs isolated by SEC showed a clear transparent appearance (Fig. 3A), while those isolated by TFF from the same serum sample assumed a more yellowish and opaque color (Fig. 3B). This was likely due to the presence of serum protein leftovers in the TFF sample. The SEM results also showed a clear difference among the two isolation methods. As presented in the representative SEM images in Fig. 3, the substrate with sEVs from the TFF method (Fig. 3F–H and S7†) showed a larger population of small particles in the size range of 10–50 nm than that obtained in the sEV sample isolated by SEC (Fig. 3B–D and S6†), for the same scanned areas.

We further estimated the sEV size distributions by counting the vesicles in a large number of SEM images. Fig. 3I and J show the histograms of the representative size distribution obtained for the two sEV samples isolated by SEC and TFF, respectively. It can be seen that these size distributions also show the same qualitative behavior for the two different isolation methods. Unlike SEC, where particle counts in the entire range of 10–200 nm were comparable, TFF showed significantly larger particle counts below the size of ∼70 nm. Furthermore, to validate our results we compared the particle distributions obtained by SEM with those obtained by NTA, the standard technique used for EV size analysis. The data clearly suggested a match between the distributions of the two methods for particles larger than 70 nm, while showing a difference in the distribution of smaller particles. As presented and supported by other reports, the SEM method accurately detected particles in the small size range (<50 nm) that could not be entirely detected by NTA or other scattering based methods.^6^ Here, we would also like to emphasize that the presence of a small difference between the two distributions, due to the characteristics of the techniques used, should be taken into account. While NTA measures the hydrodynamic radii of the particles and therefore it is likely to overestimate the size,^11^ drying techniques needed for SEM may cause a shrinkage of the vesicles, leading to an underestimation of their diameters. In addition, the thin metal layer used for high resolution imaging may also cause an overall increase of the sizes detected by SEM.

## Discussion

The importance of biophysical characterization and particularly size-based profiling of EVs has been addressed in a number of articles.^8,9,34^ Accurate size and abundance estimations of EVs may have an impact on their usefulness as a liquid biopsy source of biomarkers for cancers.^3,4,35^ A number of methods including NTA, TRPS and flow cytometry (FC) have been previously employed for this purpose. However, the results were found to be very different and technique dependent for the same EV sample.^6^ In biological samples, the variation in EV population may result in a 25 fold difference in their sizes, 20 000 fold difference in volume and 10 000 000 fold difference in scattering intensity,^6^ meaning that a number of the established methods are accurate only for a fraction of the entire EV population range. For example, size estimation by NTA and DLS relies on light scattering, which strongly depends on the particle size and material composition. In addition, interfacial properties, temperature, viscosity *etc.* also affect the results.^36^ Among the available techniques, electron microscopy still remains the most accurate approach which is also capable of covering the entire EV size range. In fact, a large number of reports on EVs have used TEM and SEM techniques but mainly as a means to visualize EVs rather than as a method for their systematic characterization and profiling. The technical bottleneck that has so far prevented these techniques from being suitable EV size profiling methods is their low throughput compared to other techniques such as NTA or FC. The results presented here aim to address this issue by methodically comparing different protocols and preparatory steps. As shown in Fig. 1E, the throughput can be significantly improved by using the covalent capture of the vesicles on the substrates and by using a suitable dehydration step (Fig. 2B–C and E–F). These approaches also improve EV distributions on the substrate, as seen in Fig. 3B. Moreover, they allow for faster analysis within a short time frame (10–15 minutes), as a sufficient number of images can be captured from different substrate areas for EV size profiling. Another advantage of the covalent strategy over the antibody-based one is that vesicles captured on substrates are representative of the whole EV population. When using antibodies instead, only the vesicle subpopulation expressing the targeted protein is captured and considered in the analysis, thus leading to subpopulation-specific size distributions.

The large measurable size range and nanometer scale accuracy of SEM also mean better reliability of the measured size distributions. As presented in Fig. 3I–J and as expected, compared to NTA, the SEM based approach shows significantly larger sEV counts in the size range below 70 nm, while closely following the NTA profile for larger diameters. This aspect has also been reported by other investigations^6,13^ and is mainly attributed to lower sensitivity and accuracy of NTA for smaller particles. In fact, depending on the preparation steps and methods, EM-based approaches are known to induce physical changes to the EV size and shapes.^32,37^ However, such effects mainly introduce an overall shift of the EVs distribution profile as all the vesicles irrespective of their sizes are affected in a similar manner. The major benefit of microscopy-based approaches is, however, their ability to accurately measure the population of the particles lying in the lower nanometer scale. As seen for the serum derived sEV sample (Fig. 3), the size distribution measured by SEM extends to very small sizes of around 10 nm and a large number of particles were detected in that range. Although sEVs are not known to exist in such a small size, it is well known that a large variety of non-EV particles, *e.g.*, protein aggregates, cell debris *etc.* may be co-isolated by the purification/isolation approach. These particles often produce significant challenges for downstream application/analysis.^38^ Their sizes are, however, far below the detection range of NTA and other scattering based approaches and therefore are not properly detected/quantified. The impurities may also include particles that have sizes similar to EVs. Previous studies have concluded that many EVs are spherical particles when in a physiological solution, while others have reported different morphologies, such as oval, cup-shaped or elongated vesicles.^39^ These morphological features of EVs should offer leverage to discriminate them from other non EV particles having random shapes, as shown in Fig. 1G and I. Our results on sEVs isolated from both cell culture medium and human serum show that the vesicles, under the imaging conditions, appear to be spherical in their morphology. However, it should be noted that the present study only involves 2D imaging at a fixed tilt of the sample stage (0 degrees) and therefore cannot be used for accurate 3D imaging. Indeed, SEM can also be used for 3D profiling, but this cannot be done with sufficient throughput to meet the objective of the present study, *i.e.*, size-based profiling of EVs. Previous studies on the morphological analysis of EVs by AFM and/or SEM reported a similar round morphology of the vesicles in the absence of an external force and that their 3D shape can be slightly flattened (diameter > height) upon immobilization on a hard substrate.^16,40^ This might also be the case in the present investigation. Overall, the SEM based approach can offer several benefits over other approaches as the morphology and size can be monitored concomitantly. This advantage enables it to be a suitable method for both size-based EV profiling and optimization of EV isolation/purification protocols. On the other hand, the size and morphology of EVs are not exclusive and therefore do not help to exclude other particles bearing the same features, such as a certain group of lipoproteins; these are lipid and protein complexes that are abundant in human blood and plasma (might account for >98% of total detected particles), thus affecting EV characterization and analyses in a liquid biopsy.^41^ Due to their morphological similarities with EVs, lipoproteins cannot be discerned solely based on their shape or size range. However, as demonstrated in this study, EVs can also be captured selectively by using antibodies targeting common EV-specific surface markers. In this non-covalent approach, one can be selective to EVs and exclude other particles bearing a similar morphology, such as lipoproteins. As mentioned above, the method can also be used for profiling (size and morphology) a specific EV sub-population, despite a decreased throughput as compared to that of the covalent EV capture method. Another way to circumvent the problem and reduce this interference can be based on the separation between EVs and lipoproteins at the sample isolation stage, before SEM imaging. This has been performed by using different techniques, *e.g.*, acoustofluidics, gel electrophoresis or magnetic beads.^42–44^ Alternatively, the use of immunogold labelling targeting EV proteins could help in distinguishing vesicles from lipoproteins during SEM imaging.^45^ In comparison, analysis by AFM can provide a wide range of information, including sub-structural organization of vesicles and mechanical properties in addition to high resolution morphological data that cannot be elucidated by the SEM approach.^16,40^ Furthermore, the need for sample fixation and/or drying and the beam-induced damage during SEM imaging can create artifacts that are not introduced when the vesicles are analyzed in liquid conditions, *e.g.*, by AFM.

Our investigation also clearly identifies different sources that can introduce artifacts which may be mistaken as EVs. These artifacts, within the scope of the present study, were mainly found to originate from the quality of reagents used and the process steps, as shown in Fig. 1A, B and S2.† The possibility of such artifacts should be carefully evaluated in the microscopic analysis of EVs. Furthermore, there have been several attempts to preserve the shape of EVs for microscopic studies in dry conditions, in particular by the use of glutaraldehyde and paraformaldehyde for fixation.^46^ Aldehyde reacts with free amino groups, stabilizing and cross linking the nucleic acid protein shell, and giving stability to the structure.^30,47^ However, as shown in Fig. 2E–F and S5,† the CPD method seemed to work quite well for preserving the morphology of EVs and additional steps with GA/PFA did not show any significant improvement in our case.

## Conclusions

In conclusion, a SEM based approach for high resolution morphological analysis and size-based profiling of EVs is demonstrated. For this purpose, we developed a protocol for improved throughput, conformity, resolution and reproducibility of EV images by comparing various preparation and imaging steps. The optimized protocol was then used to profile the size distribution of sEVs derived from human serum and also to compare the purity of two widely used EV isolation methods, *i.e.* size exclusion chromatography (SEC) and tangential flow filtration (TFF). The results revealed the presence of a higher number of small particles in the TFF sEV sample as compared to the SEC one. The size distribution profiles of these samples obtained by the proposed SEM based approach and the standard NTA based method were then compared for a qualitative assessment. The data revealed that while the distribution obtained by these two approaches closely followed each other for larger EVs (>70 nm), they significantly deviated for smaller vesicle sizes. The SEM based approach clearly identified a larger proportion of EV-like particles in the small size range (<70 nm). In addition, we also identified the presence of particles with random shapes within the reported EV sizes from the analyzed EV samples, indicating the importance of morphological analysis for accurate EV identification and size profiling. In the future, we foresee that, with some optimization, we can calibrate our SEM method to also obtain a particle count estimation from an unknown sample.

## Abbreviations

EVs: Extracellular vesicles
sEVs: Small EVs
SEM: Scanning electron microscopy
AFM: Atomic force microscopy
NSCLC: Non-small-cell lung cancer
NTA: Nanoparticle tracking analysis
FC: Flow cytometry
SEC: Size exclusion chromatography
TFF: Tangential flow filtration

## Author contributions

All authors have approved the final version of the manuscript. SC conceptualized the study, developed the protocols, functionalized the substrates, performed the pre-imaging steps, acquired the SEM images, analyzed data, and wrote the original manuscript. PH isolated NSCLC H1975 sEVs and sEVs from human serum using SEC, characterized all the sEV samples by NTA and WB, and contributed to the materials and methods section of the paper. KV supervised sEV isolation and characterization by SEC and NTA, edited and revised the manuscript and acquired funding. AK isolated sEVs from human serum using TFF and edited and revised the manuscript. KF edited and revised the manuscript and acquired funding. RL provided a research environment for the biological part and acquired funding. JL edited and revised the manuscript and acquired funding. AD conceptualized the study, wrote the original manuscript, edited and revised the manuscript and supervised the study.

## Funding sources

The supporting grants for this study were from the Erling Persson Family Foundation (to AD, JL, RL, KV and KF), Vetenskapsrådet (grant no. 2016-05051), the Stockholm Cancer Society (#171123, ##201202 (RL) and #191293 (KV)), the Swedish Cancer Society (CAN 2015/401 and CAN 2018/597 to RL), the Stockholm County Council (#20180404 to RL) and Karolinska FOUU funding (#75032 and #52614 (RL and KV)).

## Conflicts of interest

There are no conflicts of interest to declare.

## Supplementary Material

NA-003-D0NA00948B-s001
